# Syphilis Resurrected: Case Series of Palmoplantar Secondary Syphilis

**DOI:** 10.7759/cureus.46926

**Published:** 2023-10-12

**Authors:** Bushra Muna, Srikanth Shanmugam

**Affiliations:** 1 Dermatology, Mahatma Gandhi Medical College and Research Institute, Puducherry, IND

**Keywords:** acral lesions, vdrl, cutaneous syphilis, sexually transmitted infection (sti), atypical syphilis

## Abstract

Introduction

Syphilis is a sexually transmitted infection caused by Treponema pallidum which has protean manifestations. The cutaneous presentation of syphilis can mimic many dermatologic conditions.

Materials & methods

With an aim to describe palmoplantar involvement in syphilis, a retrospective study of case series was done with 11 patients having palmoplantar skin lesions in syphilis within a period of two years. Only serologically confirmed cases were included.

Results

The prevalence of palmoplantar involvement in syphilis was 47.85% and all of them except one patient (congenital syphilis) were secondary syphilis. A major proportion of cases (72.8%) studied had no history or presentation of genital lesions. Biett’s collar which is an indicator of palmoplantar syphilis was seen only in 45.5% of the cases.

Conclusion

The clinicians must be aware that palmoplantar skin lesions might be the only clinical presentation of syphilis and a high index of suspicion is needed to correctly diagnose and treat the condition in such a setting.

## Introduction

Syphilis, an ancient disease caused by Treponema pallidum showed decreased incidence in the 1990s following increased awareness among the population and better screening and diagnostic tests [1]. But, during recent times re-emergence of syphilis has been documented worldwide including in India since 2015 [2]. WHO estimates that 7.1 million adults between 15 and 49 years old acquired syphilis in 2020 [3]. 

The origin of syphilis has been postulated by the pre-Columbian, Columbian, and unitarian hypotheses dating back to 15,000 B.C. It has always been stigmatized, each country that was affected by the infection blamed the neighboring and enemy countries for the outbreak. The term ‘syphilis’ was coined by Girolamo Fracastoro, a medical personality in Verona. Later, Schaudinn and Hoffman discovered the etiologic agent of syphilis and named it Spirochaeta pallida, which they subsequently changed to Treponema pallidum [4]. 

Syphilis is transmitted by sexual contact (20% to 30% per sexual act), but it can also be spread congenitally (65%) by vertical transmission [5]. Rare methods of transmission include organ and blood donation, and inoculation as suggested by reports of extragenital syphilitic lesions on the fingers and in the nose of physicians. Also, reports of syphilis being transmitted via human bite in both sexual and non-sexual circumstances are present [6].

About 30% of patients with untreated syphilis develop irreversible cardiovascular and neurological complications. According to the Centers for Disease Control and Prevention (CDC), the rate of congenital syphilis was 48.5 cases per 100,000 live births in 2019 [7]. There are more newborns affected by syphilis than by any other neonatal infection with 520,000 adverse fetal outcomes due to syphilis annually worldwide, making congenital syphilis more common than congenital HIV infection [8]. Here, we discuss a case series of 11 cases that presented over a period of two years with palmoplantar skin lesions of syphilis.

## Materials and methods

The retrospective study of the case series was carried out by reviewing and analyzing the data of patients recorded in the departmental sexually transmitted infection (STI) register. The main objective was to detect the prevalence and describe the palmoplantar involvement in syphilis. The inclusion criteria for the study were patients of all ages and sexes who were diagnosed with palmoplantar skin lesions in serologically positive syphilis [based on Treponema pallidum hemagglutination test (TPHA)] between June 2021 to July 2023 were selected. Among 23 patients who were diagnosed with syphilis, 11 patients were selected based on the criteria of the study. Patients who were serologically negative for syphilis were excluded. The available data such as age, sex, duration, clinical presentation, treatment history, and results of serological tests were recorded, tabulated, and analyzed. High-risk behavior (HRB) such as substance abuse (alcohol, tobacco), men who have sex with men (MSM), and commercial sex workers was noted. 

## Results

Nearly half of the patients diagnosed serologically with syphilis (47.82%) were found to have palmoplantar involvement. Male predominance was noted and the median age is 35 (Table 1). The only female patient in our series was working in the healthcare sector. Except in three cases, other patients did not present or have a history of genital lesions. High-risk behavior was noted in four patients (36.3%). On partner screening, an asymptomatic female in her postpartum period was found to be positive and her infant son who is included in the series was diagnosed to have congenital syphilis with scaly plaques over extremities and hepatosplenomegaly. The most common cutaneous presentation was erythematous papules (75.7%) followed by Biett’s collarette of scaling which was seen in 45.5% of patients. On palpation, few lesions were indurated and two patients exhibited tenderness on vertical pressure. Nail involvement in the form of pitting was seen in three patients which could be due to causes other than syphilis. The venereal disease research laboratory test (VDRL) was non-reactive in two patients and weakly reactive in one patient but became positive after requesting the lab for serial dilution of the sample. TPHA test was positive in all patients and none of them were co-infected with HIV. Two patients had a history of prior treatment with injection of benzathine penicillin and doxycycline.

**Table 1:** Clinical, epidemiological and serological data of the study participants HRB: high risk behavior Key: 1-Biett's collarette, 2-Erythematous papules, 3-Hyperpigmented macules, 4-ruptured vesicles and bulla, 5-pustules

S.no	Age	Sex	HRB	Duration(days)	Type of lesion	Nail involvement	VDRL	TPHA	HIV	Genital lesions	Treatment history
1	43	M	-	7	1,2	-	NR	1:160	-	-	+
2	54	M	+	15	2	+	1:8	1:5120	-	-	-
3	28	M	-	10	3	-	1:16	+	-	+	-
4	29	M	-	21	2,3	-	1:32	+	-	-	-
5	26	F	-	20	1,2,3	+	1:32	+	-	-	-
6	32	M	-	6	1,2	-	1:16	1:640	-	-	-
7	38	M	+	30	2	-	1:64	1:250	-	+	-
8	23	M	+	20	1,2	-	1:8	1:5120	-	-	-
9	51	M	-	14	1,5	-	NR	1:2560	-	-	+
10	26	M	+	15	2,3	+	1:32	1:360	-	+	-
11	1	M	-	10	4	-	1:16	+	-	-	-

## Discussion

Syphilis, a sexually transmitted infection is classified into primary, secondary, latent, and tertiary syphilis. If the primary infection is not properly diagnosed and treated, after four to 10 weeks, the disease evolves into secondary syphilis as seen in our case series. Syphilides are eruptions of secondary syphilis presenting as asymptomatic coppery papules whose palmoplantar localization is very suggestive [9]. Among 250 patients with serologically confirmed cases in a study published from South India, only 10% were secondary syphilis while the majority were in the latent stage of syphilis and almost one-fourth of them were additionally co-infected with HIV [10]. But none of the cases in our series were positive for HIV which could be due to a limited number of cases seen or the possibility of enzyme linked immunosorbent assay (ELISA) being negative during the window period. Only 27.2% of study participants had no history of genital lesions which could be because chancre is painless and may be localized in areas that are difficult to visualize such as the rectum and vagina [11].

**Figure 1:**
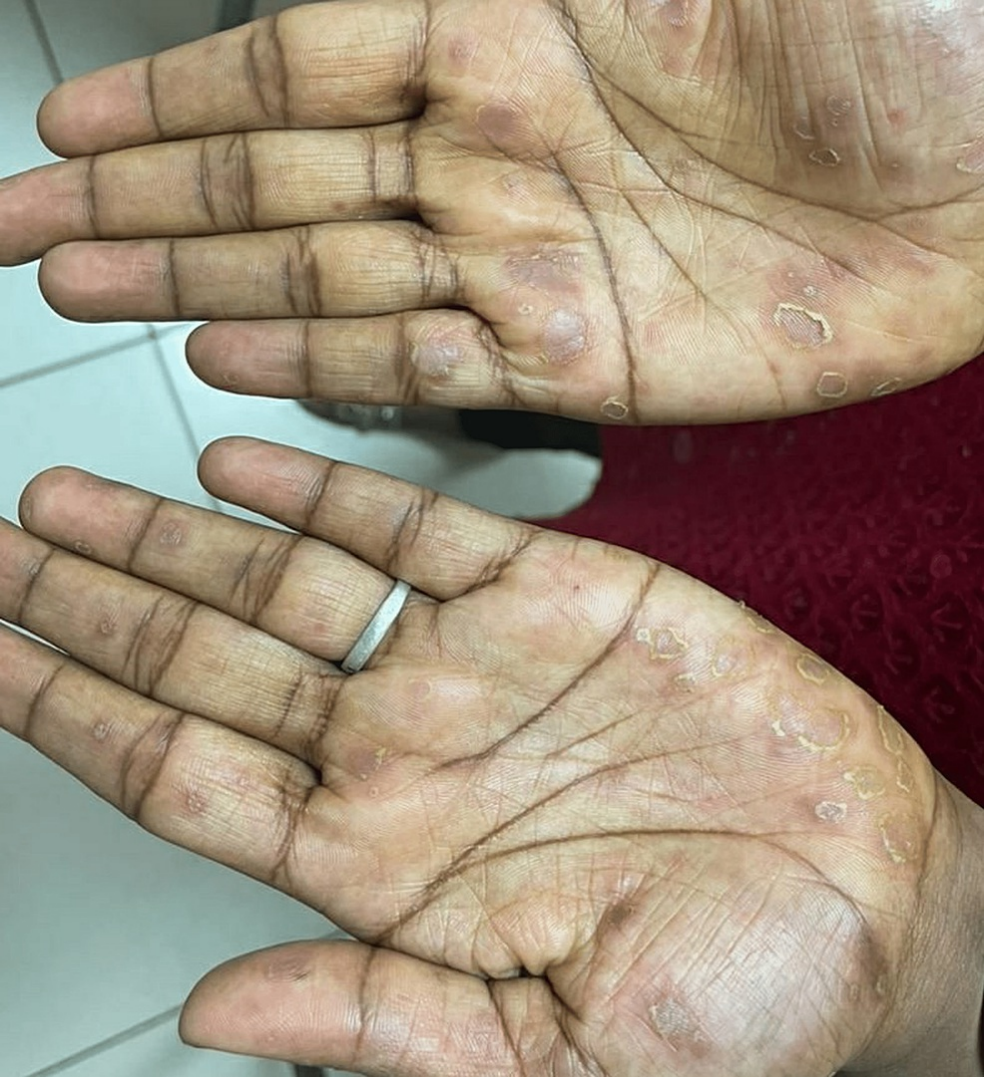
Palmoplantar syphiloderm seen as hyperpigmented macules with Biett's collarette of scales on the palms

Syphilis is famously known for being a great imitator, physicians must be familiar with all potential clinical forms of syphilis [12]. A PubMed search with the terms “palmoplantar” and “syphilis” revealed 22 results and among them, only 16 cases were reported. Within the 16 cases, only six cases had palmoplantar skin manifestations with no other clinical features of syphilis. The differential diagnosis for palmoplantar lesions of syphilis includes palmoplantar psoriasis, lichen planus, palmoplantar pustulosis, keratoderma blennorrhagicum, and hand-foot-mouth disease. The absence of psoriatic lesions in other skin areas including nails and scalp, no joint involvement, and relatively acute presentation of the cutaneous manifestations as compared to psoriasis should alert the physician to look for signs and symptoms of syphilis [13]. The presence of hyperpigmented macules with a collarette of scales - Biett's sign (Figure 1) on the palms and soles is an indicator of secondary syphilis [14]. 

Nail involvement includes nail plate changes like brittleness, splitting, fissuring, pitting, onycholysis, elkonyxis, Beau's lines, onychomadesis, and nail loss. Additionally, nail matrix change is mainly represented by syphilitic painless paronychia [15].

On dermoscopy, an orangish background is seen which is likely due to hemosiderin deposits in the dermis because of extravasation of red blood corpuscles (RBCs) in secondary syphilis. The Biett’s collar is visualized on the dermoscopy as a circular, thin, scaling edge that progresses in an outward direction and is surrounded by an erythematous halo. With dermoscopy, the evolution of syphilide is marked by the progressive extension of the Biett’s collar towards the periphery until the disappearance creating a tartar-like appearance, with a remarkable attenuation of the erythema and the orange background after one week [16]. The elicitation of tenderness on the application of blunt pressure over a syphilitic papule is the pathognomonic Buschke-Ollendorff sign which was observed in two of our patients [17]. 

A nontreponemal test like VDRL is a simple and inexpensive screening test that detects antibodies from two weeks till about six weeks after infection. Though the VDRL is 100% sensitive in secondary syphilis, it could become negative after treatment or false negative due to the prozone phenomenon. The prozone phenomenon was found to have less than 2% incidence in syphilis which could be corrected by serial dilution of the serum as seen in our case [18]. Highly specific treponemal tests like TPHA usually remain positive (85%) for the patient’s lifetime, regardless of treatment [19]. WHO recommends a combination of treponemal and nontreponemal tests for the screening and diagnosis of syphilis. The US Centers for Disease Control and Prevention recommends the approach of screening and confirmation by a nontreponemal and a treponemal assay, respectively, which was later followed by a reverse sequence algorithm. The latest recommendation by the European Centre for Disease Prevention and Control suggests screening and confirming by two different treponemal assays [20].

As per CDC guidelines, secondary syphilis should be treated by benzathine penicillin G 2.4 million units intramuscular (IM) in a single dose regardless of HIV status. In areas of high prevalence of HIV, those persons with primary or secondary syphilis with negative HIV test results should be offered HIV PrEP and retested in three months. On follow-up, clinical and serologic evaluation should be performed at six and 12 months after treatment. Re-infection and treatment failure should be considered in patients who have signs or symptoms that persist or recur and those with at least a fourfold increase in nontreponemal test titer persisting for >2 weeks [21]. Apart from the STI awareness programs and regular screening of high-risk populations, expedited partner therapy using doxycycline which is used for gonorrhea and chlamydial infection could be implemented in syphilis to prevent transmission and thereby reduce the incidence of the disease.

## Conclusions

As seen in our study, palmoplantar skin lesions could be the only sign of syphilis as the patient may not reveal the relevant history due to the sensitive nature of the information. It is imperative that clinicians know this manifestation of syphilis for the correct diagnosis of syphilis because, from a public health perspective, the secondary stage of syphilis is highly contagious, and early detection could lead to the prevention of irreversible systemic complications and further transmission of the condition. Further, physicians should be aware of the causes of false positive and false negative results of screening tests for syphilis and should take appropriate measures to aptly diagnose the disease. Small sample size, no record of dermoscopic or histopathological features, and lack of possible clinical correlation with serological titers are the limitations of this study and are the areas of need for future research.
